# Doing mental health care integration: a qualitative study of a new work role

**DOI:** 10.1186/s13033-015-0025-7

**Published:** 2015-08-22

**Authors:** Jennifer Smith-Merry, Jim Gillespie, Nicola Hancock, Ivy Yen

**Affiliations:** Faculty of Health Sciences and Menzies Centre for Health Policy, University of Sydney, PO Box 170, Lidcombe, NSW 1825 Australia; School of Public Health and Menzies Centre for Health Policy, Edward Ford Building A27, The University of Sydney, Sydney, NSW 2006 Australia; Faculty of Health Sciences, University of Sydney, PO Box 170, Lidcombe, NSW 1825 Australia

## Abstract

**Background:**

Mental health care in Australia is fragmented and inaccessible for people experiencing severe and complex mental ill-health. Partners in Recovery is a Federal Government funded scheme that was designed to improve coordination of care and needs for this group. Support Facilitators are the core service delivery component of this scheme and have been employed to work with clients to coordinate their care needs and, through doing so, bring the system closer together.

**Objective:**

To understand how Partners in Recovery Support Facilitators establish themselves as a new role in the mental health system, their experiences of the role, the challenges that they face and what has enabled their work.

**Methods:**

In-depth qualitative interviews were carried out with 15 Support Facilitators and team leaders working in Partners in Recovery in two regions in Western Sydney (representing approximately 35 % of those working in these roles in the regions). Analysis of the interview data focused on the work that the Support Facilitators do, how they conceptualise their role and enablers and barriers to their work.

**Results:**

The support facilitator role is dominated by efforts to seek out, establish and maintain connections of use in addressing their clients’ needs. In doing this Support Facilitators use existing interagency forums and develop their own ad hoc groupings through which they can share knowledge and help each other. Support Facilitators also use these groups to educate the sector about Partners in Recovery, its utility and their own role. The diversity of support facilitator backgrounds are seen as both and asset and a barrier and they describe a process of striving to establish an internally collective identity as well as external role clarity and acceptance. At this early stage of PIR establishment, poor communication was identified as the key barrier to Support Facilitators’ work.

**Conclusions:**

We find that the Support Facilitators are building the role from within and using trial and error to develop their practice in coordination. We argue that a strong organisational hierarchy is necessary for support facilitation to be effective and to allow the role to develop effectively. We find that their progress is limited by overall program instability caused by changing government policy priorities.

## Introduction

The Australian mental health system is fragmented and fails to provide coordinated and accessible services to those who need them the most. Better coordination of care that bridges the health and social care sectors has been an objective of national mental health strategies for more than two decades. There is little to show for this effort [1]. Attempts to join up care through better case management have foundered in the face of short term, highly competitive funding, which creates strong incentives against rival agencies working together.

The national Partners in Recovery (PIR) program was introduced as an attempt to build new models of coordination. PIR is designed to build new collaborative relationships to integrate mental health care from the bottom up. Its ‘partnership’ objective focuses on building new, more cooperative relationships between the non-government organizations (NGOs) which deliver much of Australia’s community based mental health care. PIR directly focuses on individuals with severe and complex mental ill-health and aims to connect the system in a way that improves the recovery of this group. Coordination relies on a new work role within the Australian mental health system, the support facilitator [2]. This paper presents an account of the experiences of Support Facilitators (SFs) in implementing this coordination, focusing in on the barriers and enablers to their work and how they have structured and conceptualised the role as they have implemented it in the first year of PIR.

The paper does not look at the PIR system as a whole, but rather focuses in on SFs as the primary ‘connecting’ element of the program. Through in-depth interviews with SFs we have sought to understand how they establish themselves as a new role in the Australian mental health system. This research takes an interpretive approach to understanding the implementation of health policy and practice. Interpretive research examines the individual sets of practices which are at the heart of any policy or practice implementation [3]. It focuses on the individuals interpreting and enacting the policy and what it means to them. This is a useful approach because a policy is only made ‘real’ by the actions of the agents who enact and actively receive it. We use their experience as a lens through which to understand the structural and practical barriers and enablers to the implementation of PIR, and specifically, the establishment of this new workforce.

## Background

### Partners in recovery

“One of the most consistent themes fed back to the Australian Government is that care for the most vulnerable people with severe and persistent mental illness is not adequately integrated or coordinated, and people with complex needs often fall through the resulting gaps.”—Department of Health and Ageing, 2012 [4].

Partners in Recovery (PIR) was developed with the dual aims of both addressing the unmet needs of people experiencing severe and complex mental ill-health and helping to connect a fragmented mental health system [2]. The program aims are to:

- provide better coordination of clinical and other supports and services to deliver ‘wrap around’ care individually tailored to the person’s needs.
- strengthen partnerships and build better links between various clinical and community support organisations responsible for delivering services to the PIR target group.
- improve referral pathways that facilitate access to the range of services and supports needed by the PIR target group and.
- promote a community based recovery model to underpin all clinical and community support services delivered to people experiencing severe and persistent mental illness with complex needs [5].

The A$549.8 million program was announced by the Gillard federal Labor government in its 2011–2012 federal budget. Implementation was subsequently delayed and reduced to $430 million over 2012–2016. Delivery was to be local and integrated with broader primary care programs using the geographical areas of the 61 Medicare Locals, the government’s new primary care organisations. Service providers, mainly from the non-government sector, formed consortia, and rival bids were launched. Only the 48 regions that submitted their proposal in the first round were funded.

**Fig. 1:**
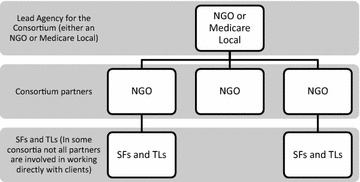
Partners in Recovery consortium structure (*SF* Support Facilitators, *TL* Team Leaders. These roles are explained below)

The PIR Consortia that emerged (see Fig. 1 below for an example) range widely in structure. Most consist of multiple NGOs—ranging from faith based national charities with substantial financial resources and policy clout, to smaller consumer-based organisations—most surviving on competitive contracts from state and federal governments. Each consortium has a lead agency, usually a Medicare Local, which handles contracts and other financial matters, manages relationships with the Department of Health and strategic direction, but plays little part in service provision [2]. All the service teams are funded from the PIR grant, but employed by various consortium members. The program has survived a change of government to the conservative Abbott Coalition government, but the four regions yet to start operations were defunded.

The use of regionally based consortia reflected a new emphasis on flexibility and locally tailored solutions to complex problems. The objectives of PIR were left vague, with much scope for local variations in implementation. They were to focus on providing ‘co-ordination of support and flexible funding for people with severe and persistent mental illness with complex needs’.

Most of the national goals focused on improving coordination across a fractured system. Local PIRs were to engage and join up the range of sectors, services and supports, build capacity through stronger partnerships, map services and identify gaps. They were to ‘engage’ with clients and stakeholders, manage referral pathways but not provide direct services themselves. The linchpins were ‘skilled Support Facilitators’ (SFs) [4].

SFs meet with individual clients in order to identify and bring to the client appropriate services and supports. Their focus is on the client’s needs with the aim of bringing together services that meet those needs and thus promote the client’s recovery. Their background training (as discussed further in the results section) is not specifically clinical with SF position descriptions variously calling for tertiary qualifications in social sciences [6], a vocational Certificate IV in Mental Health or ‘relevant experience’ [7]. While the content of the position differs slightly amongst different PIR Consortia, the basic role description sees SFs reviewing referrals, assessing client needs, and working to ‘develop and maintain partnerships’ in order ‘to meet the recovery goals of the consumer’ [6, 7]. They serve up to 30 clients each [7]. Another key role is that of the Team Leader (TL) who works alongside SFs and have a part-time SF role with a smaller case load as well as responsibility for sector development and team management.

As the program title indicates Partners in Recovery, rather than providing clinically oriented services, is charged with facilitating recovery and consciously uses a language oriented towards recovery. Recovery in this context refers to the “attainment of a meaningful, productive, and satisfying life, regardless of the presence or absence of reoccurring symptoms” [8]. Recovery-oriented practice refers to a strength-based, hopeful, client-directed approach to practice that creates an environment to best enable or facilitate recovery. It has been evidenced to be difficult for services to implement in practice because it requires a “fundamental transformation” and reconceptualisation of dominant ideologies and practices in the mental health system [9]. Recovery-orientation necessitates a focus on the client and the client’s needs with all interactions following on from that [10, 11]. Meaningfully placing the client at the centre of all interactions means that services become structured around the client rather than other structures such as diagnostic pathways, funding flows or other service imperatives. This challenges structured ways of working. As clients have needs that stretch beyond mental health services a recovery oriented approach also necessitates closer working with other sectors including housing and employment. These sectors have not traditionally seen themselves as part of the mental health system, so accessing their support on behalf of a client means making new connections.

**Fig. 2:**
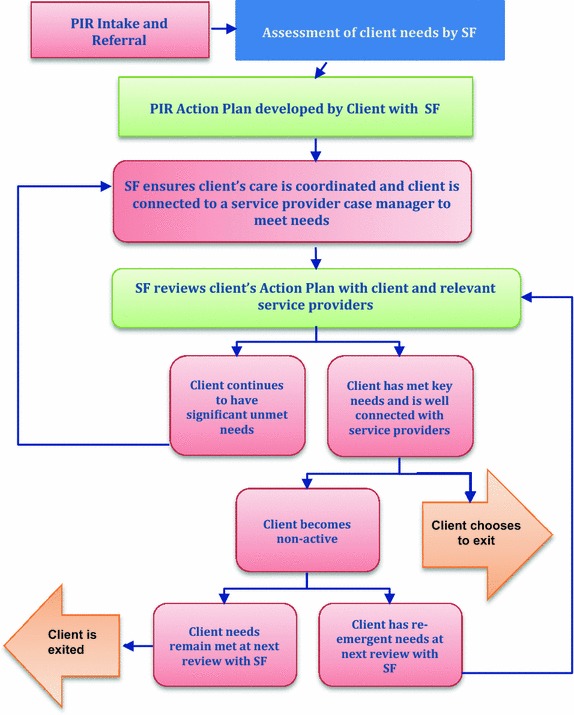
The client journey through PIR and the SF role in this journey (adapted from model used by Western Sydney Partners in Recovery)

PIR is also connected with a broader move within mental health and disability services towards a client centred commissioning model. ‘Person centred’ models have shifted to the importance of coordination of services—helping the individual find their way through a maze of fragmented health, housing and social services to build an optimum set of individually needed supports and services. The stress on ‘coordination’ and the use of flexible funding packages locate the SF within these emerging models. The national guidelines set up as PIR was established were ambiguous but distanced the eventual SF role from existing mental health professions. By the time competitive bids were called to implement the program, the job had acquired the ‘support facilitator’ tag [12]. SFs were defined as ‘coordinators’, receiving and reviewing referrals, assessing client needs, developing a PIR Action Plan around these assessed needs and engaging with existing case managers and service providers to deliver the plan. The SF role was distinguished from conventional case management and service provision. PIR related documentation repeatedly stresses that SFs are not case managers. Job descriptions, for example, specify that it is ‘not a case management role’, but SFs have a role in ensuring that the client has access to external case management which ‘is provided on a continuous basis and at an appropriate level’ to meet client needs [6, 7]. A more intensive case management role would only be offered ‘on an interim basis, with a view to establishing this function and identifying a substantive case manager early in the implementation of the PIR Action Plan’ [4]. As a ‘coordinator of the service system, not a “service deliverer” in the traditional sense’, the SF’s task was primarily to improve the system’s response to the client across their needs—building service pathways and networks of services and supports’. Substantial ‘flexible funds’ gave PIR organizations a commissioning function to purchase these services from existing providers [4]. Perhaps whether or not SF sits under a case management umbrella or not is not important. What is significant is that within the Australian context, case management or coordination type roles have traditionally been clinical in nature and embedded within government run clinical services. This is a new role within the NGO sector and as such, a new workforce within the mental health system (Fig. 2).

### History of support facilitator type roles in mental health

The term SF is not commonly used outside of the PIR setting. The only relevant use of the term that could be located in the literature was the use of the term ‘supports facilitator’ to describe a similar role working within Disability Services Queensland to support people living with mental illness who had been discharged from psychiatric hospitals into the community [13]. However, the type of role that the SF provides is not unique, with various names being given to similar roles including ‘integration coordinator’, ‘boundary spanner’, ‘care coordinator’, ‘case manager’, ‘system agent’, ‘brokerage case manager’ and ‘broker’ [13–16].

Although those who designed the SF role have consciously not used the term perhaps to distance themselves from the history of practices associated with the role (see discussion next paragraph), there is a clear link between the history of case management and the development of SFs [2, 4]. The case manager role developed from the 1970 s [14]. It was developed to specifically deal with the context of deinstitutionalisation where individuals who were moved into the community were unable to bring together or manage the services they needed [15]. Burns and Perkins [14, p.213] describe their role as “(1) Assessment of client needs. (2) Development of a comprehensive service plan. (3) Arrangement of service delivery. (4) Monitoring and assessment of services. (5) Evaluation and follow-up.” These initially non-clinical roles were deemed to lack necessary clinical knowledge and were replaced by case managers who had clinical expertise [14, 17]. These ‘clinical case managers’ have been typified by those working in Assertive Community Treatment. Other clinical case managers focus their practice on psychosocial rehabilitation and the strengths model [14]. More recent work has asserted that clinical skills are not necessary to the case management role [16]. Different levels of case management have been identified [16]. The simplest is ‘brokerage’ which includes assessment, care plan development, arrangement of care, evaluation of care provided and maintenance of contact with the client [Holloway in 15]. Other levels of case management offer direct clinical support or offer recovery oriented support by focusing on a client’s skills and abilities e.g. ‘strengths case management’ [15]. The brokerage framework equates most closely with support facilitation in the context of PIR. Support facilitation within PIR includes case-finding, assessment of needs, service finding, evaluation of needs met and case closure but does not extend to the more intensive roles of clinical management or psychosocial support, although these needs will be identified and clients referred to appropriate services if these are needs expressed by the client.

There has been a move away from the use of terms ‘case manager’ and ‘case management’ because of concerns that the role is not fulfilling its original intent or is ineffective in meeting its intent even if implemented faithfully. Reviews of case management have highlighted that it is not generally effective in meeting its aims, although Marshall et al. [15] provide an exception for case management structured around a ‘strengths’ model (which is aligned with recovery). Rapp and Goscha [18] also highlight the effectiveness of the strengths approach but state that the form of case management with the least positive impact for clients is the broker model (which most closely aligns to PIR) with only two of the nine studies reviewed showing a benefit. In Australia Brophy et al. [2] point to the Survey of High Impact Psychosis to demonstrate that the current system of case management in Australia can be seen as patchy at best for those in the target group of PIR. They also highlight problems related to the terminology which positions people as ‘cases’. This is not aligned with a recovery-oriented approach to mental health.

### New work roles

Of the considerable research on new work roles in health there is little that focuses on either mental health or the introduction of new roles from a sector rather than a workplace perspective. Despite this there are some learnings from the literature which are useful in informing our understanding of the implementation of the SF work role in Australia. Leading with a good overview of the literature Maxwell et al. [19] examined the introduction of new nursing roles in NHS hospitals in the UK and found that acceptance of the role depended on acceptance of a shared social identity between the new role and existing roles. The literature shows that the introduction of new work roles is not always successful with problems arising from poor relationships, poor role definitions, a lack of demonstrable achievement and competition from existing professional boundaries [19–21]. Recent work focusing on the introduction of peer support roles within mental health care and support settings perhaps offers most in our understanding of the challenges [22–24]. These roles are both recovery focused and new to the mental health system but differ significantly in type of role.

## Method

This paper presents results from a secondary analysis of a sub-set of interview data collected during late 2013 and 2014. The broad aim of the interviews was to gain an understanding of the implementation of PIR with respect to its goal of increasing the connectedness of the fragmented mental health system. The interviews were part of a larger set of 41 interviews with PIR stakeholders and consortium members to provide an initial view of the implementation of PIR across the Western Sydney region. These interviews were transcribed and thematically analysed. The secondary analysis reported in this paper was conducted separately and focused on the support facilitator’s role and experience.

### Interviews

Interviews were conducted in two PIR regions in Western Sydney. These two regions together serve a population of 1,439,259 people and have a total estimated population of 4190 that are eligible for PIR support (PIR aimed to meet the needs of 40 % of this population) (Department of Health and Ageing, 2012). As at June 2015 the two regions have a combined total of 688 clients enrolled in the program. In total, 11 Support Facilitators (35 % of all SFs in the study regions) and 4 Team Leaders (36 % of TLs in regions) were interviewed across the two PIR programs. Interviews with TLs were included in the analysis as in these regions they have a reduced SF client load and facilitate the work of SFs.

### Process

Ethics approval was gained from the University of Sydney Human Research Ethics Committee. Relevant organisations were identified with the aid of a research reference group consisting of PIR consortium members, stakeholders and consumers. Researchers contacted these relevant organisations to ask them to participate in interviews. They then identified possible respondents within their organisations. All organisations and respondents contacted agreed to be interviewed. As interviews progressed further organisations to interview were identified where they were mentioned in multiple interviews or particular gaps in our understanding of the field emerged. Individual in-depth, semi-structured interviews were conducted face-to-face. All participants provided informed written consent prior to the interview. The average interview duration was 40 min. Interview questions aimed to get an overall picture of system connectedness at an early stage of the program implementation. Opening questions focused on the role and background of both the individual interview respondent and organisation they belonged to before moving on to questions about knowledge sharing, enablers to connectedness and barriers to connectedness. Interviews were audio recorded and transcribed verbatim.

### Analysis

The focus of this research is on the establishment of the SF as a new role in the Australian mental health system and a set of research questions was devised which sought to explore this process. The SF and TL interviews were therefore analysed according to the questions in Box 1 below.

**Box 1:** Research questions for secondary data analysis on SF and TL roles in system connectedness

Overall research question:
How do Support Facilitators establish themselves in the system?
Sub questions:
1. What does their daily work consist of?
2. How do they conceptualise their role?
3. What enables their work?
4. What are the barriers they face in their work?

We conducted an inductive thematic analysis of the data where the data were coded through an open coding process based on the research questions. A second coding reduced the number of codes and determined the main themes relevant to answering the research questions. This coding was checked against a sub-section of coding conducted separately by another member of the research team and alignment of coding confirmed.

Representative quotations are used to bring alive the data using the SF or TL’s own words. Quotations are identified by alphanumerical identifier and numbers of respondents reporting a particular idea is presented in brackets e.g. (3) in order to demonstrate the extent to which participants presented a particular view. This adds to the validity of the findings as the reader is able to assess the extent to which the views presented here are widely held by the respondents.

While the analysis was structured around the four sub-research questions listed in Box 1 above it made more sense to structure the discussion of the results below according to the core themes identified in the analysis as the main themes that emerged stretched across the different research questions.

## Results

### Background knowledge held and used by Support Facilitators

SFs described coming from a variety of training, work and volunteering backgrounds including nursing, psychology, homelessness services, disability, psychology, policy, refugee advocacy and social work (13). These backgrounds give them a broad range of skills that are “based on a position description” rather than qualifications [SF4]. When directly asked about what knowledge they drew on in their work most did not mention training but rather spoke about ‘experience’ or ‘own knowledge’ or mentioned previous work roles [SF2; SF8]. They commented on the diversity of backgrounds in order to demonstrate the richness of experience amongst their teams. From the SF and TL perspective, the divergent educational and experiential knowledge that was being drawn on was seen as an advantage: “It is a great cross-section of people from lots of really interesting backgrounds. I like that. I think we draw a lot from each other in terms of backgrounds and skills…” [SF5] The diverse backgrounds were viewed as a valuable resource for individuals in allowing them to understand problems from different perspectives and to access contacts established in previous roles (11): “I’ve worked for different programs and I’ve also known people from different programs, so it’s good for me to say, hey, I’m so-and-so, can you help me with this client? They will help me if there is a spot.” [SF7] Several SFs stated that own personal experience was valuable for the role (5) including their own experience of mental ill-health and as carers for family members. While this personal experience was seen as a valuable asset for their position, the SF role is not specifically a peer support role.

In contrast to the otherwise consistent SF view that their diversity of backgrounds was an asset, two respondents commented that a lack of clinical experience could also present a barrier to their acceptance in the field, especially from clinical mental health services, for example: “I’ve been asked my background and my qualifications. I feel like saying pole dancer.” [SF5] or “…you go into a clinical team and say, I’m from Partners in Recovery and they go what are your qualifications? You say why is that relevant? I’m not diagnosing anybody. I’m not medicating anybody. I am supporting them.” [TL1].

### What does the daily work of Support Facilitators consist of?

The daily work of the SFs centres around meeting and connecting with most time reported as being spent in individual meetings; attendance at interagency meetings; SF forums; site visits; online connecting and committee meetings. SFs meet face to face with PIR clients in order to establish their needs and follow up on whether these are being met or have changed. They also meet with fellow SFs and Consortium members through intra-agency meetings and work to establish interagency connections. These connections were made primarily in order to get referrals (of new clients) and make referrals (for current clients). The process of making and concreting connections was thus the most pressing task that SFs dealt with in their jobs and at the heart of their work. This is discussed in detail below.

#### Interagency connections

The most frequently cited types of forum were interagency meetings such as those dealing with homelessness and drugs and alcohol dependency (12). Interagency meetings were described as bringing together various organisations involved in a particular area to share information about difficulties, successes, needs and so forth. For example, “we’re talking to lots of different services…all these places that have the same thing happening, working with consumers that have issues with hoarding and squalor and about how they’re supported.” [SF3] Participants explained that some interagency meetings focus on a particular place or client group and all those services who deal with that group will attend, for example, “It’s a combined inter-agency, so you’ve got age mental health, housing, pretty well everything” targeting a particular locale [SF2]. SFs reported using these interagency meetings to: get services to refer clients to PIR; make contacts which they could then use to find services for their clients; and to educate the sector about the role of SFs and PIR (12). The interagency meetings were therefore key to the functioning of PIR and core tools for developing broader understanding of the role of SFs.

Strategies were developed to manage and make best use of interagency forum involvement by the SFs. One TL respondent stated that there were “44 or 45 different interagency forums that we became aware of that were happening in the [area], different focuses of each forum and different people being invited and that kind of stuff.” [TL2] To cover all of these interagency forums the SFs and TLs then created a system so only one SF was involved in each of the relevant forums. These people would then feed back into working groups of SFs who had a particular interest in that area:

“So working groups, for example, there’d be three, four, five people who have a real passion for disability, physical disability, intellectual disability …. Disabilities is just one example. Education and employment, alcohol, drugs, Aboriginal services et cetera.” [TL2].

“…so we’ve kind of been dividing up into working groups and particular interest areas. So been going to the Child and Family, DV [Domestic Violence] and LGBT [Lesbian Gay Bisexual and Transgender], they’re the … working groups that I’ve been dividing those interagency meetings up.” [SF8].

“…the physical health working group, we’ve been talking about different health prompts like, [named organisation] has a health prompt that we use with consumers, so I’ve shared some information about that to the group and we’ve received information from other organisations.” [SF4].

These working groups therefore operate as ways to channel into the consortium knowledge from the broader sector and consolidate knowledge being collected by SFs in their interactions with clients.

#### Intra-agency connections

Another commonly cited forum for sharing knowledge were groups involving all the SF and TLs in a PIR region (5). These forums are used for SFs to share knowledge about a particular topic, or to invite in a guest speaker to provide information about a topic relevant to the work of PIR. Take for example the following quotations:

“…every month or so we’ve had each of the host agencies organising presenters from the different services that are out there to come in and speak with the wider team…. [or] a wider team meeting where all of the Support Facilitators and perhaps the team leaders as well can share some information or have discussion about what’s going on and what are the areas of concern.” [TL2].

“Yeah it is very helpful because in that forum sometimes they invite services like Centrelink, could be Housing or…the psychological team to come and give us information about a specific topic.” [SF1].

The ability to draw on fellow SFs’ background knowledge, connections and developing knowledge about the field was the key driver for the establishment of SF and TL forums for sharing knowledge.

The SFs also present client cases in the forums. They did this for two main reasons. First, to illustrate or exemplify the work that they were doing with a particular population (e.g. hoarders [SF3]), and second to draw on the knowledge of the assembled group in assisting their work with a particular client [SF7,SF10]:

“…basically if someone’s a bit stuck with someone they can just bring that client and everyone can contribute and see what we can come up with…. So this way if someone’s really stuck on something they have somewhere to take it and they’ve got a room full of people who are passionate.” [SF10].

“…we do have Support Facilitators meeting every month and we share good stories and difficult… We can talk to people and see what type of client challenges they’ve got and what type of service challenges they’ve got. We could be learning thinking, oh we work in a different way, but this is all right too…. there was comparison and also there was learning there too.” [SF7].

SFs and TLs use these meetings to assist each other to meet individual client needs, but also to learn and change practice based on the experiences of other SFs, building together shared effective practices for service delivery.

Free flowing communication via phone and email was viewed as particularly important to the effectiveness of the SF role (10). One SF spoke about the importance of “open communication and using all the communication tools. So…using the phone—sometimes just texting even—email–fax; whatever it takes to communicate with everyone.” [SF3]. This was mainly used for working with existing networks, contacts or working groups in order to track down information to help clients: “We just ring each other if we’re stuck for a service and we’ll send out an email saying ‘hey, I’ve got this, I’m looking for a great female GP in the [local] area, does anyone know of one?’…people will look and see if they have a contact that they’ve already worked with” [SF6]. One TL stated that quick flow of communication was so important that they consciously chose to fit out their SFs with the best tools for communication available: “…we’re equipping our Support Facilitators and our team with laptops and mobile phones and iPhones and that kind of stuff” [TL2].

Conversely poor communication was identified as a significant disabler to the work of the SF (3). One TL stated that they felt “frustrated” when they could not provide effective communication supports or knowledge flows for the SFs [TL2]. Communication was so integral to the SF role that their work in connecting could not be done without effective communication taking place.

#### Connections for clients

Respondents emphasised the great variability in their practice which resulted from the personalisation of services to clients [TL1, TL2, SF8, SF9, SF4, SF6]. ‘Connecting’ work centred on the individual experiencing mental ill-health. Every interaction was viewed as “individual with the client” [SF9], necessitating flexibility and resourcefulness. This variability seemed to be accepted as a natural part of the job. Only one respondent complained about the variability of their work creating difficulties [SF8].

While the importance of client-focused connections was emphasised by six respondents, there were only a few mentions of typical office-based client meetings. Interactions with clients were not structured around set office or home visits and could be informal [SF1,SF6, SF7,SF9]. As SF9 described: “Taking a holistic view as we do to a person means collaborating with a really wide range of services, agencies, and—maybe not services…. like shop owners or something. You go and walk with a client, think about how their mornings are going to be spent every day…. It’s a really important part of addressing someone’s sense of being themself and belonging.” Other SFs recounted trips to Centrelink [the national employment agency], a GP office and a medical specialist appointment.

### How do Support Facilitators conceptualise their role?

#### Development of a collective Support Facilitator role

Six respondents emphasised the collective nature of SF work. SF2 spoke about the role of SF being something that they “build” in the ‘talk’ and through the development of a collective SF team: “we talk about everything. We share…. It is developing that team…”. While SFs hold and use their individual knowledge which they draw on to make connections they also work as a team with other SFs and TLS across the consortium and this greatly enhances their work through the sharing of experience and knowledge:

“It’s SF, team leader knowledge and networking and sharing information essentially. So we have a very large breadth of knowledge across the whole [team]…. So they all bring with them that knowledge… and you share that and so the kind of the depth of knowledge has just developed”. [TL1].

“…we work together as a team, so it’s a combination of drawing on the collective experience of [our] colleagues…” [SF11].

“the other knowledge that we do draw on is a reliance, I suppose, on everyone. Share their knowledge.” [TL4].

This peer support between SFs who worked at “sharing and encouraging each other” [TL3] was viewed as key to the development of the role and its success in the field.

Three respondents went so far as to speak about a shared identity between all SFs in their region. For example: “…all the SFs are there for the consumers and we’re all there because we want the best outcomes for these people. So we’re more than happy to share our knowledge and experience with each other.” [SF6].

#### Working with clients

In finding services for clients SFs drew on multiple connections, for example those made through their previous work (8),the connections held by their organisation (3), their fellow SFs (discussed in the next section below) or current clients. Three respondents spoke about their interactions with current clients helping to extend the PIR network and develop links useful to other clients [SF9,SF4, SF8]. Focusing on the client was thus also a means of extending the PIR network: “…you have an individual and then because of that individual you might then talk to four or five different services. That I suppose opens up those conversations to further working.” [TL1] All of the contacts were viewed as important because they extended the PIR network and may be useful for future clients or in getting new referrals to PIR. A good relationship with current clients could even lead to referrals through that client:

“I had one consumer who I ended up getting through a consumer advocate who had worked with me with another consumer, and had kind of been talking about Partners In Recovery, and then had convinced another consumer who didn’t really want support, that we were okay and we weren’t so bad.” [SF8].

Surprisingly, given the name of the program, only two respondents spoke about recovery-orientation as a named framework structuring their interactions with clients [SF3, SF4]. SF4 spoke about their PIR practice as a type of educational tool to teach both the individual client and the rest of the sector about recovery: “…we’re sort of modelling to other organisations how we like to work and how we think best works and that—empowering the consumer to take a lead in their own recovery.” Others, while not expressing their practice in terms of recovery-oriented practice, described engaging in person centred approaches. In all 11 of the 15 respondents spoke about their work being client focused in that they acted according to the specific needs of their clients. SFs put a great deal of effort into understanding the needs of their clients “…because it takes a long time to build trust and respect from people who’ve been let down by the system for so many years…” [SF6]. One also spoke about learning from their client, which narrowed the gap between them: “I just took myself into that journey, thinking, what would myself be? I may not have been that strong like her, without a single cent in my account, no husband, no kids. I started thinking and then I understood more and I learned more…” [SF7] As these quotations illustrate a recovery orientation was being evidenced amongst these SFs even when it was not named as such. A core facet of recovery oriented service delivery is the breaking down of hierarchies between staff and clients [11]. These quotations evidence a levelling out of the client staff relationship. However there was no evidence of SFs considering recovery from the client’s perspective, or speaking about the recovery journey of PIR clients.

### The challenges and barriers faced by Support Facilitators

Respondents articulated a range of barriers to their work. The most consistently reported barriers related to communication difficulties and confusion or resistance in the service community about what PIR has to offer.

#### Identity, acceptance and role clarity

SFs spoke about the difficulties associated with being in a role that was new to the system [SF3; SF5; SF8]: “I think one of the challenges could be that it’s a very particular type of role that you do that hasn’t—it’s not something that you can kind of go, oh yeah we’re a case manager, or oh yeah—it doesn’t fit…” [SF3] They expressed difficulty in getting those outside of PIR to understand this role and in differentiating it from existing roles in the system: “I think some of that’s been based on role, a sense of we’re doing the same thing, so what are you doing that we’re not.” [SF8] Getting their clients to understand the limits of this new role also meant that they sometimes went beyond its limits: “So then it’s very easy to turn into a support worker. I try really hard not to, but sometimes you are like look someone is trying really hard to do something. I’m going to take them—for example to their appointment because it’s raining and they’re near…” [SF5] The role of a support worker is about intervention by the worker and therefore has a different role to a support facilitator.

#### Communication

Poor communication was viewed as a significant barrier to SF work. This can be seen as natural given the great importance on communication for establishing connections to bring in client referrals and find services to work with PIR clients. Areas of communication difficulties were identified between the central PIR consortium management [SF2; SF5; SF9; TL2] and SFs or between the stakeholder organisations and PIR [SF2, TL1, SF5, SF9].

### Communication challenges

The following quotations illustrate the difficulties and frustrations created by a lack of internal PIR communication:

“I think unless we get… the lead agency working, we just can’t work because it all just filters down and it’s a nightmare. Then what you’re starting to get now is Support Facilitators who are restless and frustrated and bored.” [SF5].

“PIR’s about services and communicating better with each other to make the system work better. If we can’t [do that] in an organisation then how the hell are we going to change the system if we’re not communicating.” [SF2].

Some of the difficulties related to confusion about measurement and data collection, including of performance, quotas and client outcomes [TL3; TL2; SF9; TL4]. SFs wanted to be told if they were “making a difference” [TL2] or to understand what the data they were collecting would actually be used for. Other communication difficulties were around referrals, which were causing “bottlenecks” in the operation of the system [SF4] and changing messages from the lead agency about processes to be followed [SF10].One SF illustrated these difficulties in the following way, articulating the messiness caused by a lack of strong coordination from the Lead agency:

“…we’re all on a canvas, but why’s the canvas unclear, so fragmented?” [SF9].

The nationally defined structure of the PIR consortium means that while SFs work and have their contracts of employment within individual organisations, the format of their work follows rules set by the local PIR consortium (see Fig. 1, above). This means that they are subject to direction from two separate sources and must comply with policies and directions from both. One respondent stated that this could cause inconsistency in the way different SFs were able to operate with some having to follow more restrictive organisational policies that others did not [SF4]. At the same time, SFs benefited by drawing on the existing connections between the organisation and the sector in making referrals. However, SFs in some organisations felt that they were disadvantaged in their work compared to those SFs working in organisations that had a larger ongoing presence in mental health work in their areas. One TL spoke about competition between the SFs in different NGOs “We are being put in competition with each other. It’s been like oh, you’ve done this and you’ve got five stakeholders. Why aren’t you doing that?” [TL3] This competition was seen to be fed from the lead agency in the consortium with “negative feedback” turning the SFs “into competitors against each other” [TL3]. However one of the TLs directly contradicted this description of competition stating “I actually thought it was going to be very difficult—enemies and competitors—and it’s not even there.” [TL4], instead highlighting the competition between the SFs and the lead agency. While these quotations contradict each other, what they do highlight is that lead agency communication strategies created barriers between members of the consortium.

Communication difficulties with stakeholders was also a barrier to the work of the SFs. The reasons were variously given as confusion or scepticism about PIR, a history of competition in the sector, stigma and process difficulties internal to the stakeholder organisation. Respondents pointed out poor channels for communication to stakeholder organisations (for example could not ever speak to the person they needed to) [SF9], or other operational issues effecting the organisation. One respondent recounted an interaction with a stakeholder which included non-response, wrong documentation, repeated requests for further information, lost referrals, silence and then a final interaction: “called them a month later and they said there’s a 6-month waiting list and that’s because of PIR.” [SF5].

Confusion or “scepticism” [SF8] about PIR was mentioned by five respondents. SFs described feeling that PIR was viewed by others as lacking credibility because it was a new program in the sector [SF3]. The struggle for SFs then was in “having other people know what we do, and getting that credibility about how we do it and what we’re doing and the point of it. The job of SF is very different to other jobs [SF3].” One respondent stated that “trying to have those conversations and sort of gain an understanding of what PIR does has been really difficult in that area, in my opinion.” [SF4] Part of the difficulty could be, as discussed earlier, the “attitude of an individual service” [TL1] and resistance to what was deemed as PIR’s incursion into another organisation’s territory:

“We’re trying new things, they hate us because we are like yeah we are going to do this and they are like no you are no, we’ve been working with this person for 10 years. So it’s really convoluted and grey and very messy.” [SF5].

For this reason SFs described needing to be ‘strategic’ in their approaches to the field [SF9]. Confusion was also expressed about the distinctive contribution of PIR “There are a lot of services out there, so filtering—having PIR being filtered through the noise of those other services so that they are the ones that people think of [is] going to be a challenge” [SF11]. Constant outreach involving presentation of PIR methods and goals was seen to be the key to “break through that noise” [SF11].

Respondents reported that it was particularly difficult to make contact with certain types of service for example GP practices or clinical services [TL2, SF9, SF4]. This was either because they were isolated or resistant to PIR operating within a traditionally clinical setting: “Clinical services don’t respect us [because] we’re an NGO.” [SF5] Conversely non-clinical services which SFs needed to make contact with to address a client’s non-clinical needs could be resistant to working with PIR because of stigma towards working with people experiencing mental ill-health [SF5, SF6, SF9].

Historic competition in the sector around funding was another disabler to the connecting work of SFs. While one respondent commented that the collaboration through PIR and other joint collaborative projects was breaking this down, it was still something that stopped effective communication and connections being made: “…historically a lot of these NGOs, a lot of these services [are] competing for tenders, competing for funding, it’s intellectual property, we want to look after ourselves and make sure [we do] all the capacity building ourselves and that kind of stuff.” [TL2] Another respondent emphasised these intellectual property concerns “…people can think that you’re trying to see what they’re doing in their work… that you’re making a judgement.” [SF3] These historical divisions which had separated the system were thus a significant barrier to SF attempts to join it up again.

## Discussion and conclusions

The overall aim of this research was to understand how SFs worked to establish themselves as a new work role in the existing mental health and social support sectors. The research questions were deliberately broad and aimed to get at the experience of SFs in order to understand their interpretation of the role. Reflection on these experiences as presented in the data gives rise to further thinking about both the SF role and the system in which it is situated.

While the SF role is not a ‘new role’ in that it bears a very strong resemblance to the brokerage model of case management, it is a new role in the context in which it is being applied. The PIR’s SF role represents a large and expanding, new workforce within the Australian mental health sector and unlike previous case management roles, is non-clinical and embedded in the NGO sector rather than within clinical service delivery systems as has traditionally been the case. New roles entering existing settings are inevitably faced with challenges related to the need to fit into an already functioning system (however broken the system might actually be) [23]. This is compounded when there is a lack of preparation as the new roles appear as if dropped out of thin air [21]. This was the case with PIR. Implementation was hasty, spurred by (accurate) fears that the Labor government would lose power at the end of 2013. As a result, PIR was thrust on the mental health sector and the Medicare Locals with little preparation. Workforce planning is usually only something that occurs within an individual organisation or professional grouping [25]. Where the new role needs to work across a sector and lacks a clear professional background, as with PIR, those occupying the new role are given the task of planning and defining its scope and preparing the sector for their arrival. This ‘preparation’ work can be seen in the presentations SFs and TLs give to the sector and the constant networking associated with the role. Uncertainty about the boundaries of a new role by either the SF or those outside PIR mirror the experiences of other new roles establishing themselves within sectors, including peer support workers [23]. The SFs are educating the sector as they work and selling themselves and the PIR program they represent in every interaction. This makes these interactions crucial to the success of the entire program and makes difficult the interactions at the edge of traditional boundaries, as evidenced in conflict from some areas, and difficulties in making contact with others (e.g. GPs).

SFs are not just educating the sector about their role but are engaged in a process of understanding and educating themselves. Banfield et al. [1] make the point that descriptions of the process of ‘coordination’ in Australian health policy and implementation planning documents, including mental health, are so vague as to be meaningless. PIR is no different and SFs must work out how to ‘do’ the process of coordinating and connecting while involved in it. They are involved in a process of trial and error: testing what works, where difficulties lie and how to get around them. While the background knowledge SFs brought with them was significant, the embodied knowledge gained through on the job experience was emphasised as more important. This pattern is consistent with conventional understandings of how practitioners learn to do their work [26] through ‘on the job’ experiential learning rather than structured training. These results show the key to this learning process is communication between SFs. The interactions through forums, collaboration and one–one–one meetings develop a common service model, but also develop a shared way of being a SF, or shared SF *practice*. Communication is thus key to an effective support facilitation role. Using the work of Freeman [27] we can see that they are “learning by meeting” with the micro-interactions of the meetings building personal and collective practice. Shepherd and Meehan [13] further point to interpersonal communication as “the ‘glue’ of interagency collaboration”. However without effective and open networking this type of communication becomes problematic. As our respondents so clearly document, the SFs and others working across the sector were able to sort themselves into informal ad-hoc but pragmatic working groups and forums. These things cannot be forced but, as the SF experience shows, specific support for informal networking is essential through funding, role descriptions, and management practices which need to be loose enough to allow the groupings to emerge.

Previous research has highlighted that strong internal governance arrangements from the host organisations are essential for the success of both case management and new roles. Brophy et al. [2] emphasised that PIR could not be successful without strong management of each local consortium, with clearly defined roles and responsibilities for each partner organisation. Lack of strong central organisational support from within the PIR consortium was highlighted in our interviews as a significant difficulty for SFs in their work in implementing PIR. This may be part of the inevitable teething problems of a new program, but should be addressed as a core concern for PIR consortia. The next round of interviews with the same respondents at 1 year post-baseline will determine whether this is an ongoing issue for PIR. It may be that the lack of consortium coordination reflects a lack of guidance by the Federal Department of Health under a new conservative government hostile to legacy programs started under Labor. The Medicare Locals, which provide the lead agency for most consortiums, have been abolished and replaced with weaker ‘Primary Health Networks’ with considerably less autonomy and with a geographical scope which in many cases no longer corresponds with PIR consortium boundaries.

The manner of introduction of new work roles into an existing system has an impact on its acceptance within the sector. Ross et al. [16] write that case manager type roles will be most successful when they are implemented within a system characterised by “shared vision and objectives, close links between health and social care, aligned financial flows and incentives, stakeholder engagement [and] provision of services in the community”. SFs only have control over one aspect of these conditions. The experiences of SFs and TLs exposes some of the tensions and difficulties which led to the fragmented system that PIR is in part meant to fix. The interview responses reveal a system which is still fragmented and the effects of years of perverse incentives to collaborative working (many of which continue) which will take more than just a new job role to fix. As Whiteford et al. [28, p.904] comment: “The standout issue needed to promote effective service integration is arguably the hardest to achieve; that is, ensuring mutual respect and understanding of roles with streamlined communication between all the services involved in the care and support of clients”. High level decisions about funding and collaborative service development above all need more stable funding models. Even with the best will on all sides, the interviews reveal a reluctance by many stakeholders to invest too much in the relationship with PIR as its funding expires in 2016. Stakeholders likely understand that nothing has changed in the funding and organisation of the sector beyond PIR meaning that the competitiveness which divided the sector prior to PIR and which the SFs work against will continue post-PIR.

Given that the PIR program expressly mentions recovery in its title and has a stated aim of enhancing recovery for individuals experiencing severe and complex mental health it was surprising that only two of the respondents mentioned the recovery concept in the interviews. Evidence of recovery orientation in the interviews was only partial, and did not include important elements including a focus on self-management, use of a strengths model, promotion of citizenship, involvement where appropriate of family and care giver support people or engagement of clients with peer support [29, 30]. Interviews showed no evidence of attempts to promote recovery in PIR clients or of an aim to understand recovery from a client’s perspective. However this may reflect the interviewing than SF practices because the interview questions did not ask SFs about their interactions or progress with clients. Other reasons may be that the recovery concept is so ingrained in their work that there was little need to mention it, or that recovery is not being actively spoken about and promoted within PIR consortia. If the latter is true this is a significant deficit as the client and communication focus of the SF role means that it is perfectly placed for promoting recovery within the sector and reorienting the system around recovery values. A lack of a deep understanding of recovery from the client’s perspective may also be a symptom of a workforce new to mental health. In both sites recovery training has since been provided to staff and follow up interviews may therefore demonstrate improved recovery orientation of SFs. One-third of the respondents in these interviews stated that they drew on their own experience of mental ill-health, including as a client or carer. This is an important finding because it demonstrates the value of lived experience as a resource for those employed in SF roles.

This study was conducted within two metropolitan regions and results therefore only reflect the experiences of SFs and TLs working in cities. The experiences of SFs working in regional areas may be different. Further research is needed which draws on the local evaluations in regional areas in order to understand support facilitation in those contexts. Another limitation of this study is that we are only reporting an understanding of the SF role during the early stages of program implementation. However, our evaluation of PIR will also include interviews with the same organisations at 12 months (data collection currently in progress) and 24 months so we will be able to understand how the role develops over time. This is important because new roles will generally change over time as will the challenges and enablers they face [21]. Data collection at 12 months and 24 months with PIR stakeholder will also provide data which reflects on the SF role from an external perspective, and therefore provide another view of the role.

Our findings on SFs have relevance beyond PIR. Programs such as the National Disability Insurance Scheme (NDIS) in Australia and Paths to Personalisation in the UK necessitate an increasing role for service facilitators and brokers. Close working between PIR SFs and National Disability Insurance Agency (NDIA) workers has taken place in the NDIS trial site in the Hunter Region of New South Wales. NDIS uses a similar brokerage model of case management and is likewise being implemented as a new overarching program in existing settings as it is rolled out throughout Australia. While the future of PIR is uncertain it is likely that it will either merge with NDIS or complement it closely. Innovations such as personal health budgets and other types of person-centred care practices mean that there are increasing spaces to be filled by ‘accented’ SF type roles in, for example, managing money and buying in, finding or negotiating access to services [18]. Efficient systems through which these innovations can be implemented will only be possible if we understand what it means to work in these roles and what helps them to negotiate the system.

## Abbreviations

Respondents are identified through alpha-numerical identifier. Support Facilitator (SF) respondents are identified by SF1, SF2 etc. and Team Leaders (TL) by TL1, TL2 etc. Numbers in brackets e.g. (11) refer to how many respondents made a specific point. PIR means Partners in Recovery. NDIS means National Disability Insurance Scheme.
